# Diagnosis of Multiple Organ Dysfunction in Neonates with Hypoxic–Ischemic Encephalopathy: Vasoactive Inotropic Score, Renal Score, Fibrosis-5 Index and Lactate/Albumin Ratio

**DOI:** 10.3390/diagnostics14242796

**Published:** 2024-12-12

**Authors:** Başak Kaya, Hasan Akduman, Dilek Dilli, Nilden Ünsal, Nurdan Dinlen Fettah, Ayşegül Zenciroğlu

**Affiliations:** 1 Department of Neonatology, Dr. Sami Ulus Maternity and Child Research and Training Hospital, Babur st., Number: 36, Altındag 06080, Turkey; akduman2004@yahoo.com.tr (H.A.); dilek.dilli@sbu.edu.tr (D.D.); nurdan.dinlen@sbu.edu.tr (N.D.F.); aysegul.zenciroglu@sbu.edu.tr (A.Z.); 2 Department of Pediatrics, Dr. Sami Ulus Maternity and Child Research and Training Hospital, Babur st., Number: 36, Altındag 06080, Turkey; drnilden@gmail.com

**Keywords:** hypoxic–ischemic encephalopathy, organ dysfunction, fibrosis-5 index, vasoactive inotrope score, prognosis

## Abstract

Background: Vasoactive inotrope score, renal score, fibrosis-5 index, and lactate-albumin ratio have not been investigated before in determining multiple organ dysfunctions accompanying infants with hypoxic–ischemic encephalopathy (HIE) in neonatal intensive care units (NICUs). The aim of this study was to determine whether multiple organ dysfunctions that may accompany HIE in infants are correlated with vasoactive inotrope score (VIS), renal score (RS), fibrosis-5 index (FIB-5), and lactate-albumin ratio (LAR), and whether these parameters can predict morbidity and mortality. Methods: This is a retrospective study, and 106 newborns diagnosed with HIE and treated with hypothermia were included in the study. Vasoactive inotrope score for cardiac dysfunction, renal score for renal dysfunction, fibrosis-5 index, and lactate/albumin ratio for hepatic dysfunction were evaluated. Results: We found that the vasoactive inotrope score, renal score, fibrosis-5 index, and lactate-albumin ratio values of infants diagnosed with HIE are associated with cardiac, renal, and hepatic dysfunction. These values, calculated on the 2nd postnatal day, are particularly linked to prolonged hospital stay and mortality, which are key prognostic factors. Conclusions: Our study is the first to combine vasoactive inotrope score, renal score, fibrosis-5 index, and lactate-albumin ratio parameters in determining organ dysfunction in newborns with hypoxic–ischemic encephalopathy and to reveal their prognostic and mortality prediction values. Therefore, although it offers new perspectives, new studies are needed.

## 1. Introduction

Hypoxic–ischemic encephalopathy is a heterogeneous clinical condition characterized by peripartum or intrapartum hypoxic events and neurological dysfunction in the infant. The incidence of HIE is between 1 and 2 per 1000 full-term births in developed countries and is more common in developing countries [1].

Although therapeutic hypothermia (TH), which is the standard treatment for HIE, has been proven to provide positive results in terms of survival and neurological development in infants, HIE continues to be one of the most important causes of mortality and morbidity in NICUs. [2,3]. Initiating TH as early as possible in infants diagnosed with HIE has been shown to increase neuroprotection and reduce seizure burden, especially in moderate HIE [4,5,6]. There are two methods that can be used in TH: one is selective head cooling and the other is whole body cooling. Studies have not shown any significant difference in 12-month neuromotor development or mortality rates between selective head cooling and whole-body cooling in newborns with HIE. Although superiority over the other method has not been demonstrated, whole body cooling is a more preferred method with more experience [7]. While the initial TH protocols included infants with moderate to severe HIE, the study (PRIME) on infants with mild encephalopathy found that these infants had abnormal amplitude electroencephalography (aEEG) or seizures at discharge, abnormal brain magnetic resonance imaging (MRI) or abnormal neurological examinations, disability at 19 months of age, low Bayley III scores, and abnormal neurodevelopmental outcomes [8,9,10,11].

TH also leads to favorable outcomes in HIE, but there is a significant relationship between the severity of HIE and multiple organ dysfunction and mortality. Neurological dysfunction observed in infants with HIE is a serious complication of hypoxic–ischemic injury and may be accompanied by multiple organ dysfunctions that further contribute to morbidity and mortality [12]. Determining the relationship between these multiple organ dysfunctions and HIE severity and analyzing how they are related to each other can guide clinical judgment and allow for early intervention [13,14]. Among these analytical methods, clinical scoring systems that provide information about the prognosis of the disease have been utilized. Sweetman et al. evaluated multiorgan dysfunction in patients with neonatal encephalopathy using the multi-organ dysfunction scoring in neonatal encephalopathy (MODE) scoring system, which assesses the cardiovascular, respiratory, gastrointestinal, hematological, and neurological systems. They reported that MODE scores were higher in infants with moderate/severe neonatal encephalopathy [15]. Toptan et al. found that Hemoglobin-Albumin-Lymphocyte-Platelet (HALP) scores were higher in newborns with HIE who developed seizures, periventricular leukomalacia (PVL), and kidney injuries [16].

In our study, unlike in previous studies, we aimed to determine the organ dysfunctions of vasoactive inotropic score, renal score, fibrosis-5 index score, and lactate/albumin ratio, which have never been evaluated in infants with HIE, and to investigate whether each of these parameters can be used specifically in that organ failure. The secondary aim was to evaluate the relationships of these parameters with the prognosis and mortality of infants with HIE.

## 2. Materials and Methods

### 2.1. Study Design

In this study, 153 newborn babies who were diagnosed with HIE and received TH treatment in our hospital’s (Dr. Sami Ulus Maternity and Child Research and Training Hospital Ankara, Turkey) neonatal clinic between 2017 and 2023 were identified. Of these babies, 42 were excluded from the study because their clinical data were insufficient, three were excluded because TH was terminated before 72 h, and two died within the first 24 h. As a result, the study was continued by analyzing the data of 106 babies. Ethics committee approval was obtained from our hospital (AEŞH-24.01.2024-056). HIE was diagnosed when the baby’s history of acute intrapartum/peripartum hypoxic event was accompanied by an APGAR score of <5 at the 5th and 10th minutes, pH < 7.00 or BE < −12 in cord blood gas or blood gas values taken within one hour after birth, brain damage consistent with HIE on MRI or MRI Spectroscopy, and multiple organ failure or involvement were detected [17]. The Modified Sarnat score was used to determine the HIE stage by evaluating clinical findings [18]. The HIE diagnostic criteria, modified Sarnat score, and aEEG monitoring of the babies included in this study were evaluated and the decision to start TH treatment as soon as possible was made under the responsibility of the neonatal intensive care specialist. TH was applied in the form of whole-body cooling within the first six hours after birth to late-term or term babies (≥36 weeks) diagnosed with HIE. Close monitoring of Stage 1 HIE cases that we thought did not fully meet the TH treatment criteria was performed with aEEG monitoring (for basic brain activity and seizure detection), and TH was started before the six hours after birth in cases with aEEG abnormalities. TH was applied by keeping the core temperature measured at 34.5 °C with a probe inserted into the rectum for 72 h, cooling the whole body, and then slowly increasing the body temperature (0.5 °C/h) to 37 °C in the following 6 h. During therapeutic hypothermia, dexmedetomidine was administered to the infants for sedation and inotropic agents for bradycardia.

### 2.2. Clinical Data

The demographic characteristics (gestational age, birth weight, APGAR scores, gender, and mode of delivery) and clinical characteristics (HIE staging with Modified Sarnat scoring: Stage 1, 2, 3, system dysfunctions, length of hospital stay, and mortality rates) of the included infants were obtained from medical records.

Central nervous system dysfunction was evaluated using aEEG, clinical seizures, conventional EEG, MRI, Diffusion MRI, and MRI Spectroscopy. Cardiorespiratory system dysfunction was assessed through pulmonary hypertension defined by echocardiography, increased serum creatine kinase-myocardial band (CK-MB) and troponin I values, and hypotension with the need for inotropic support. Respiratory failure was identified using the need for mechanical ventilatory support, surfactant deficiency due to hypoxia, and the presence of meconium aspiration syndrome. Gastrointestinal-hepatic system dysfunction was assessed based on aspartate aminotransferase (AST) and alanine aminotransferase (ALT) levels > 100 U/L, albumin levels < 2.7 g/dL, and/or coagulopathy secondary to liver dysfunction international normalized ratio (INR) > 1.5, activated partial thromboplastin time (APTT) > 50 s, prothrombin time (PT) < 70% and/or hemorrhagic diathesis), as well as the presence of feeding intolerance and necrotizing enterocolitis. Renal dysfunction was defined as decreased urine output (<1 cc/kg/h), elevated or rapidly increasing creatinine levels (>1.5 mg/dL or a 0.3 mg/dL increase within 24 h), and thrombocytopenia with platelet levels < 100,000/mm^3^. Data obtained retrospectively from the hospital automation system were recorded on patient study forms.

Using the clinical and laboratory data from the hospital’s computer system, the vasoactive inotropic score, renal score, fibrosis-5 index score, and lactate-albumin ratio values were calculated, and results for the first, second, and third postnatal days were recorded. The associations between these parameters and the patient’s clinical findings were analyzed. The formulas for these parameters are provided below [19,20].

**Vasoactive Inotropic Score:** Dopamine dose [μg/kg/min] + dobutamine dose [μg/kg/min] + 100 × epinephrine dose [μg/kg/min] + 10 × milrinone dose [μg/kg/min] + 10,000 × vasopressin dose [U/kg/min] + 100 × norepinephrine dose [μg/kg/min])

**Renal Score:** Difference between peak creatinine value in the first 72 h and baseline creatinine value) × 10

**Fibrosis-5 Index Score:** (Albumin [gr/dL] × 0.3 +Platelet count [10^9^/L] × 0.05) − (Alkaline phosphatase [U/L] × 0.014 + AST/ALT × 6 + 14)

**Lactate-Albumin Ratio:** Lactate (mmol/L)/Albumin [gr/dL]

### 2.3. Statistics

All data were analyzed using SPSS 26 (IBM SPSS Statistics for Windows, version 26.0). Descriptive statistical analysis (mean + standard deviation, percentage) was applied to demographic data. The Kolmogorov–Smirnov test was used to control the normality of the distribution of quantitative values. According to Kolmogorov–Smirnov, quantitative variables were expressed as mean ± SD or median (min-max). We applied the Mann–Whitney U-Test and correlation analysis to evaluate the relationship between VIS, RS, FIB-5, and LAR numerical values and clinical findings. According to clinical findings, the etiology of inotropic support requirement was evaluated in three groups (those who did not need inotropic support, those who needed inotropic support due to hypotension, and those who needed inotropic support due to bradycardia) and the Kruskal–Wallis Test, a nonparametric test, was applied to measure the relationship between inotropic support requirement and VIS, RS, FIB-5, and LAR numerical values and mean quantitative variables of three or more groups. In case of Kruskal–Wallis Test results *p* < 0.05, the Mann–Whitney U test was used as post hoc test. In addition, ROC curve analysis was used to obtain the cut-off value for numerical parameters (VIS, RS, FIB-5, and LAR). A two-sided *p*-value of <0.05 was considered statistically significant. We found a significant statistical relationship between VIS and cardiac involvement, RS and renal dysfunction, FIB-5, LAR and hepatic dysfunction [respectively: *p*: <0.01, correlation coefficient: 0.8; *p*: <0.001, correlation coefficient: 0.72; *p*: 0.048, correlation coefficient: 0.2; *p*: 0.007, correlation coefficient: 0.5].

## 3. Results

The demographic and clinical data of the study patients are presented in Table 1. The mean gestational age of all patients was 38.7 ± 1.4 (36–41) weeks. According to the Sarnat criteria, 52 patients (49%) were classified as Stage 2–3 HIE, while 54 patients (51%) were classified as Stage 1 HIE. Advanced resuscitation, including cardiopulmonary resuscitation and intubation, was required for 10 babies (9.4%) diagnosed with HIE in the delivery room. Additionally, the 5 min APGAR scores of babies in the Stage 2–3 HIE group were significantly lower compared to those in the Stage 1 HIE group (*p*: 0.002). The mean time to start hypothermia treatment was 4.88 ± 3.8 h (0.5–24).

The mean hospitalization period for infants with Stage 2–3 HIE was 23.8 ± 38.4 days (5–120 days), and the mortality rate was 9.6%, which was significantly higher compared to the Stage 1 HIE group (*p*: 0.006, *p*: 0.003). In infants with Stage 2–3 HIE, the LAR was higher, and the FIB-5 was lower (*p*: 0.016, *p*: 0.02). A statistically significant relationship was found between the elevation of VIS, RS, and LAR and the decrease in FIB-5 calculated on the postnatal second day and mortality (respectively, *p*: 0.011 correlation coefficient: 0.4; *p*: 0.003, correlation coefficient: 0.30; *p*: 0.03, correlation coefficient:0.32; *p*: 0.006, correlation coefficient: −0.24). In the ROC analysis for predicting mortality, the area under the curve and optimal cut-off values were 0.976 and ≥18.7 for VIS, 0.954 and ≥7.6 for RS, 0.908 and ≥23.2 for LAR, and 0.781 and ≤−32.3 for FIB-5, respectively.

When evaluating infants for cardiorespiratory dysfunction, 50 infants were found to have respiratory dysfunction and 75 infants had cardiac dysfunction. Among the 50 infants with respiratory dysfunction, 28 required both invasive and noninvasive mechanical ventilation support (4/28 infants received surfactant treatment for respiratory distress syndrome, and 14/28 had pulmonary hypertension), while 22 received only noninvasive mechanical ventilation support (5/22 infants received surfactant treatment for respiratory distress syndrome). For the 75 infants with cardiac dysfunction, inotropic agent support was initiated in 28 due to hypotension (10/28 had pulmonary hypertension) and in 47 due to bradycardia (4/47 had pulmonary hypertension). In infants with high VIS, there was a significant relationship between the presence of pulmonary hypertension, the need for invasive mechanical ventilation, central nervous system involvement, prolonged hospital stays, and mortality (Table 2). Out of the infants evaluated for renal function, 34 (32%) had renal dysfunction. The RSs of infants with renal dysfunction was significantly higher compared to those without renal dysfunction [*p*: <0.001, correlation coefficient: 0.72 RS:0.82 (0.54–1.00)]. A high RS was statistically significantly associated with cardiovascular and gastrointestinal dysfunction, prolonged hospital stays and mortality (Table 2). A statistically significant association was found between high RSs and cardiac dysfunction (those receiving inotropic agent support) (*p*: 0.03). The RSs of infants who started inotropic support due to hypotension were higher than those of infants who started inotropic support due to bradycardia [*p*: 0.018] (Table 2). Of the infants evaluated for hepatic dysfunction, 41 had hepatic dysfunction. The FIB-5 values of infants with hepatic dysfunction were statistically significantly lower, and LAR values were significantly higher than those without hepatic dysfunction [*p*: 0.048, correlation coefficient: 0.2, cut-off value: −28.6 (−37.5…−19.6) for FIB-5; *p*: 0.007 correlation coefficient: 0.5 cut-off value: 7.2 for LAR]. A significant relationship was found between low FIB-5 values and the need for intubation after cardiopulmonary resuscitation in the delivery room, the requirement for ≥2 antiepileptic treatments due to clinical seizures, and mortality. High LAR was significantly associated with cardiorespiratory system dysfunction, gastrointestinal system dysfunction, central nervous system dysfunction, prolonged hospital stays, and mortality (Table 2). Of the 41 infants with hepatic dysfunction (51.2%), 21 had coagulation disorders.

## 4. Discussion

In this study, we found that the VIS, RS, FIB-5, and LAR values of infants diagnosed with HIE were correlated with cardiac, renal, and hepatic dysfunction. We found that these values calculated on the 2nd day after birth were associated with prognostic factors, especially long hospital stay and mortality. Cardiovascular dysfunction in HIE is attributed to hypoxia. Metabolic acidosis and multiple organ damage due to hypoxia vary according to the severity of the condition [21]. Cardiac involvement in HIE negatively affects myocardial contractility, cardiac output, and blood pressure. Pulmonary hypertension is common. In addition, therapeutic hypothermia applied in the treatment of HIE affects hemodynamic function by causing bradycardia, peripheral vasoconstriction, and decreased cardiac output [22,23]. Hypotension and bradycardia may lead to secondary multiorgan ischemic damage. Systemic hypotension and bradycardia treatments include dopamine, dobutamine, adrenaline, and noradrenaline. Cardiovascular dysfunction is assessed by various methods, including clinical findings, biochemical parameters, echocardiography, and other hemodynamic assessments. In this study, we assessed cardiovascular system involvement using the VIS, which combines clinical, hemodynamic, and echocardiographic assessments. There was a statistically significant association between high VIS and the presence of pulmonary hypertension, the need for invasive mechanical ventilation support, central nervous system involvement, the need for fresh frozen plasma support, prolonged hospital stays, and mortality.

The frequency of respiratory dysfunction in infants with HIE ranges from 23% to 86% [24,25,26,27]. This dysfunction may range from transient supplemental oxygen requirements to severe, persistent pulmonary hypertension. In our study, respiratory dysfunction was observed in 50% of infants with HIE. However, due to the retrospective nature of our study, we were unable to comprehensively evaluate the ventilation scores of our patients. Scoring systems such as the inotropic score and VIS have established tools for predicting clinical outcomes in the pediatric intensive care unit [28,29]. Recent studies have shown that vasoactive ventilation renal scoring serves as a powerful predictor in the postoperative period [30,31]. Mileric et al. found that vasoactive ventilation renal scores influenced morbidity and mortality outcomes in infants with postoperative congenital heart disease [28,30]. Alam et al. reported that median VVR scores exceeded concurrent median VISs and that VVR scores consistently provided a better predictor of clinical outcomes than VISs [30]. Sherer et al. found that VVR predicted prolonged intensive care unit stay more accurately than lactate and VIS values in the early postoperative period [32]. VVR score was independently associated with prolonged duration of mechanical ventilation (>96 h) in newborn infants undergoing cardiac surgery [33].

In our study, we suggested that VIS and RS parameters assessed for the first time in infants with HIE may more accurately reflect organ-specific dysfunction. We observed that high VIS and high RS were associated with prolonged hospital stay and increased mortality. Renal dysfunction and acute kidney injury are common complications of neonatal encephalopathy and contribute significantly to morbidity and mortality. Although there is no definitive treatment for acute kidney injury, early recognition and management of fluid, electrolyte, and metabolic imbalances and understanding the underlying pathophysiology can potentially reduce associated morbidity [34]. Acute kidney injury occurs in HIE with a frequency ranging from 22% to 70% [35]. Selewski reported that infants with acute kidney injury secondary to HIE have longer hospital stays and mechanical ventilation [36], and Sarkar et al. reported that infants with acute kidney injury have abnormal brain MRI findings [37]. Creatinine is not an ideal biomarker for neonatal acute kidney injury because it peaks late, rises only when 50% of renal function is impaired, may reflect maternal levels, and indicates renal function rather than injury. The KDIGO guidelines include increased creatinine as part of the definition of acute kidney injury [38]. However, many studies still rely on serum creatinine and urine output to diagnose acute kidney injury [39]. Our retrospective study found a significant association between renal dysfunction and renal score, with 32% of HIE patients showing renal involvement. We showed that high RS was significantly associated with cardiovascular and gastrointestinal dysfunction, prolonged hospital stays, and increased mortality.

Shah et al. [24] defined hepatic involvement in HIE as elevated AST and ALT levels >100 IU/mL within the first-week post-birth. Hankins et al. observed transaminitis in 80% of HIE infants, defined as levels 1.5 times the upper limit of normal. They suggested that elevated lactate dehydrogenase, ALT, and AST levels to 1.5 times the upper limit of normal indicate liver involvement in HIE [26]. In our study group, which included infants with HIE, LDH elevation was universally present. Choudhary et al. demonstrated that the severity of HIE is associated with increased ALT and AST levels, though abnormalities in hepatic synthetic function markers like albumin and prothrombin were not linked to HIE severity [40,41]. The FIB-5 score has not been previously evaluated in studies including pediatric patients. There were studies on FIB-4 in pediatric patients. Leung et al. reported that AST to platelet ratio index was better than FIB-4 for predicting cystic fibrosis liver disease and severe cystic fibrosis liver disease [42]. Chen et al., in a study conducted to detect fibrosis in patients with choledochal cyst, found no significant difference between the aspartate aminotransferase (AST)-platelet ratio index and fibrosis-4 index (FIB-4) [43]. Initially used as a marker for hepatic damage and fibrosis in adults, the FIB-5 score has since become a critical risk classification tool for predicting cardiac events in acute heart failure and cases of heart failure with preserved left ventricular ejection fraction [20]. Okamoto et al. found that patients with acute decompensated heart failure and low FIB-5 scores faced a high risk of death or rehospitalization [44]. Eto et al. linked the FIB-4 and FIB-5 indices with poor stroke outcomes [45]. Wang et al. observed that, in evaluating acute cardiac and cerebral events, low FIB-5 scores combined with high C-reactive protein levels were associated with a poor prognosis [46]. In our study, we found that fibrosis-5 index accurately reflects liver involvement in HIE and found a correlation between low FIB-5 score and hepatic dysfunction. We also found a significant association between low FIB-5 score and cerebral involvement (such as aEEG abnormalities, clinical seizures, and antiepileptic drug use) and mortality.

Lactate, a byproduct of anaerobic metabolism, reflects tissue hypoperfusion. However, elevated lactate levels can be influenced by various factors including seizures, cardiac arrest, liver dysfunction, metabolic disorders, medications, diabetic ketoacidosis, and burns [47]. Impaired liver function affects lactate metabolism, leading to its accumulation. Conversely, albumin, produced by the liver, is a marker of liver function. Elevated lactate levels or reduced albumin levels indicate liver dysfunction, making the LAR a more accurate reflection of liver metabolism. Studies have shown that LAR is a valuable predictor of mortality in heart failure patients [48]. Kabra et al. reported that LAR provided excellent prognostic value in predicting sepsis outcomes, with 100% sensitivity and 88% specificity, outperforming lactate and albumin alone [49]. In 2020, some researchers proposed that LAR might be an early prognostic marker for intensive care unit patients and could predict mortality [46]. Nishioka et al. identified LAR as an independent predictor of high in-hospital mortality in heart failure patients post-myocardial infarction, suggesting it was superior to lactate and SOFA scores [50]. Shi et al. found that high LAR at intensive care unit admission was an independent risk factor for both short-term (30-day) and long-term (360-day) all-cause mortality in patients with acute kidney injury [51]. Wang et al. also noted LAR’s association with various conditions, including traumatic brain injury [52]. Overall, LAR has proven clinically significant in critically ill patients, such as those with heart failure and acute kidney injury [53,54]. Although studies in children are rare, Arı et al. highlighted LAR’s significance in predicting mortality in children with hospital acquired infections at 24 h postnatal [55]. Aygün et al. found that L/A ratio (>0.5) was associated with mortality in critically ill children [56]. Our study also found that low FIB-5 score and high LAR were associated with hepatic dysfunction. We observed a statistically significant relationship between high LAR and high RS in infants with HIE (*p* < 0.001, correlation coefficient: 0.45). Additionally, high LAR was significantly associated with cardiorespiratory system dysfunction, gastrointestinal system dysfunction, central nervous system dysfunction, prolonged hospital stays, and mortality.

Although our study offers new perspectives as it is the first study to include VIS, RS, FIB-5, and LAR parameters in newborns with HIE and to show their prognostic and mortality predictive values, it is limited by its retrospective nature and relatively small sample size obtained from a single-center experience. One of the limitations of this study is that we were unable to assess the long-term prognostic impact of the organ dysfunction parameters we assessed in the study. Another important limitation is that biomarkers with prognostic significance in detecting acute kidney injury at an early stage in a critically ill neonate population could not be assessed due to the retrospective nature of our study. The most suitable of these biomarkers for our study was neutrophil gelatinase-associated lipocalin in urine and serum. Neutrophil gelatinase-associated lipocalin is associated with the severity of HIE and can predict subsequent creatinine increases [57]. Urinary neutrophil gelatinase-associated lipocalin levels were significantly higher in neonates with moderate-severe HIE than in infants with mild HIE [58]. Identifying organ dysfunction in infants with HIE, and particularly in acute kidney injury, which is a common complication, is important to improve long-term outcomes and enable early intervention. There is a need for prospective studies to evaluate both the use of new biomarkers in the diagnosis of organ dysfunction parameters and the effect of organ dysfunction parameters on long-term prognostic results.

## Figures and Tables

**Table 1:** Demographic and Clinical Characteristics of the Study Patients.

Characteristics	
Maternal age (yr) (mean ± SD), (min-max)	28 ± 5.4 (18–40)
Gestational week (mean ± SD), (min-max)	38.7 ± 1.4 (36–41)
Gestational weight (grams) (mean ± SD), (min-max)	3181 ± 445 (2000–4660)
Gender (male), *n* (%)	67 (63.2)
Delivery type (Cesarean section), *n* (%)	42 (39.6)
Gravida 1, Parity 1	55 (51.8)
Gravida 2, Parity 2	24 (22.6)
APGAR score (mean ± SD), (min-max)	
1st minute	3.65 ±1.7 (0–9)
5th minute	6 ± 1.7 (1–10)
10th minute	6.7 ± 1.7 (1–10)
HIE stage	
Stage 1 *n* (%)	54 (%51)
Stage 2–3 *n* (%)	52 (%49)

**Table 2:** Relationship between fibrosis-5 index, lactate/albumin ratio, renal score, vasoactive inotrope score parameters, and clinical findings.

	Lactate/Albumin	Fibrosis-5 Index	Renal Score	Vasoactive Inotropic Score
	*p* Value			
**HIE Stage (Modified Sarnat stage)**				
**Mild (Stage1)** **Moderate-Severe (Stage1–2)**	0.016 *	0.02 *		
**Cardiovascular system**				
Echocardiographic evaluation (At the first 24 h) Pulmonary hypertension Mitral insufficiency	0.01 *		0.013 * 0.028 *	0.017 *
CK-MB level (Normal <25 U/L) >250 U/L	0.002 *		0.006 *	
Troponin I level (Normal <0.037 ng/mL) >0.037 >0.12	0.03 * -	- 0.03 *	- 0.015 *	
Hypotension (with inotropes) Bradycardia	0.02 * 0.001 *		0.03 * 0.018 ** (0.013 *)	
**Respiratory system**				
Cardiopulmonary resuscitation in the delivery room	0.02 *	0.039 *	0.05 *	
Respiratory distress syndrome	0.02 *		0.016 *	
Invasive mechanical ventilatory support	0.05 *		0.068 *	<0.001 *
**Central nervous system**				
aEEG disorder		0.04 *		
Conventional EEG	0.04 *			
Clinical seizure		0.06 *		
Antiepileptic treatment (≥2 drugs)	0.04 *	0.05 *		0.011 *
Central nervous system imaging (finding of hypoxic effect) Transfontanel ultrasonography Diffusion magnetic resonance imaging	0.002 *		0.001 *	0.012 * 0.012 *
**Gastrointestinal system**				
Liver dysfunction (AST and ALT > 100 U/L)	0.007 *	0.048 *	0.002 *	
TDP support	0.001 *		<0.001 *	0.048 *
Crio support	0.02 *		0.01 *	
Vitamin K support (>2)	0.035 *		0.02 *	
Platelet support	0.03 *		0.022 *	
**Prognosis**				
Prolonged hospital stay > 2 week	0.022 *		<0.001 *	0.04 *
Mortality	0.006 *	0.03 *	0.003 *	0.011 *

* Mann–Whitney U-Testi ** Kruskal–Wallis Testi (post hoc test).

## Data Availability

The data presented in this study are available on request from the corresponding author. The data are not publicly available due to [there is no address/link available for authors to access the data and the data was obtained from the hospital computer system].

## References

[1] Greco P., Nencini G., Piva I., Scioscia M., Volta C.A., Spadaro S., Neri M., Bonaccorsi G., Greco F., Cocco I. (2020). Pathophysiology of hypoxic-ischemic encephalopathy: A review of the past and a view on the future. Acta Neurol. Belg..

[2] Wu L., Chang E., Zhao H., Ma D. (2024). Regulated cell death in hypoxic-ischaemic encephalopathy: Recent development and mechanistic overview. Cell Death Discov..

[3] Huntingford S.L., Boyd S.M., McIntyre S.J., Goldsmith S.C., Hunt R.W., Badawi N. (2024). Long-Term Outcomes Following Hypoxic Ischemic Encephalopathy. Clin. Perinatol..

[4] Gunn A.J., Thoresen M. (2006). Hypothermic neuroprotection. NeuroRx.

[5] Thoresen M., Tooley J., Liu X., Jary S., Fleming P., Luyt K., Jain A., Cairns P., Harding D., Sabir H. (2013). Time is brain: Starting therapeutic hypothermia within three hours after birth improves motor outcome in asphyxiated newborns. Neonatology.

[6] Low E., Boylan G.B., Mathieson S.R., Murray D.M., Korotchikova I., Stevenson N.J., Livingstone V., Rennie J.M. (2012). Cooling and seizure burden in term neonates: An observational study. Arch. Dis. Child. Fetal Neonatal Ed..

[7] Celik Y., Atıcı A., Gulası S., Okuyaz C., Makharoblıdze K., Sungur M.A. (2016). Comparison of selective head cooling versus whole-body cooling. Pediatr. Int..

[8] Prempunpong C., Chalak L.F., Garfinkle J., Shah B., Kalra V., Rollins N., Boyle R., Nguyen K.-A., Mir I., Pappas A. (2018). Prospective research on infants with mild encephalopathy: The PRIME study. J. Perinatol..

[9] Chalak L.F., Nguyen K.A., Prempunpong C., Heyne R., Thayyil S., Shankaran S., Laptook A.R., Rollins N., Pappas A., Koclas L. (2018). Prospective research in infants with mild encephalopathy identified in the first six hours of life: Neurodevelopmental outcomes at 18–22 months. Pediatr. Res..

[10] Murray D.M., O’Connor C.M., Ryan C.A., Korotchikova I., Boylan G.B. (2016). Early EEG grade and outcome at 5 years after mild neonatal hypoxic ischemic encephalopathy. Pediatrics.

[11] Conway J.M., Walsh B.H., Boylan G.B., Murray D.M. (2018). Mild hypoxic ischaemic encephalopathy and longterm neurodevelopmental outcome—A systematic review. Early Hum. Dev..

[12] Chong W.H., Ong H.Y., Ooi J.S., Eleen Khaw Y.Y., Lim L.M., Tew M.M., Koo H.W., Aishah A.R., Goh P.W. (2024). The effect of hypoxic ischemic encephalopathy towards multi-organ complications and its early outcome at a Malaysian district hospital. Med. J. Malays..

[13] Michniewicz B., Al Saad S.R., Karbowski L.M., Gadzinowski J., Szymankiewicz M., Szpecht D. (2021). Organ Complications of Infants with Hypoxic Ischemic Encephalopathy Before Therapeutic Hypothermia. Ther. Hypothermia Temp. Manag..

[14] Alsina M., Martín-Ancel A., Alarcon-Allen A., Arca G., Gayá F., García-Alix A. (2017). The Severity of Hypoxic-Ischemic Encephalopathy Correlates With Multiple Organ Dysfunction in the Hypothermia Era. Pediatr. Crit. Care Med..

[15] Sweetman D.U., Strickland T., Isweisi E., Kelly L., Slevin M.T., Donoghue V., Meehan J., Boylan G., Murphy J.F.A., El-Khuffash A. (2022). Multi-organ dysfunction scoring in neonatal encephalopathy (MODE Score) and neurodevelopmental outcomes. Acta Paediatr..

[16] Toptan H.H., Tezel K.G., Tezel O., Ataç Ö., Vardar G., Gülcan Kersin S., Özek E. (2024). Inflammatory and Hematologic Liver and Platelet (HALP) Scores in Hypothermia-Treated Hypoxic-Ischemic Encephalopathy (HIE). Children.

[17] Committee on Fetus and Newborn (2014). Hypothermia and neonatal encephalopathy. Pediatrics.

[18] Sarnat H.B., Sarnat M.S. (1976). Neonatal encephalopathy following fetal distress. a clinical and electroencephalographicstudy. Arch. Neurol..

[19] Ozturk E., Tanidir I.C., Gunes M., Genc S.B., Yildiz O., Onan I.S., Haydin S., Guzeltas A. (2020). The effects of Vasoactive-Ventilation-Renal score on pediatric heart surgery. North. Clin. Istanb..

[20] Maeda D., Kanzaki Y., Sakane K., Tsuda K., Akamatsu K., Hourai R., Okuno T., Tokura D., Nakayama S., Hasegawa H. (2022). Prognostic value of the liver fibrosis marker fibrosis-5 index in patients with acute heart failure. ESC Heart Fail..

[21] Armstrong K., Franklin O., Sweetman D., Molloy E.J. (2012). Cardiovascular dysfunction in infants with neonatal encephalopathy. Arch. Dis. Child..

[22] Wood T., Thoresen M. (2015). Physiological responses to hypothermia. Semin. Fetal Neonatal Med..

[23] Gebauer C.M., Knuepfer M., Robel-Tillig E., Pulzer F., Vogtmann C. (2006). Hemodynamics among neonates with hypoxic-ischemic encephalopathy during whole-body hypothermia and passive rewarming. Pediatrics.

[24] Shah P., Riphagen S., Beyene J., Perlman M. (2004). Multiorgan dysfunction in infants with post-asphyxial hypoxic-ischaemic encephalopathy. Arch. Dis. Child. Fetal Neonatal Ed..

[25] Martin-Ancel A., Garcia-Alix A., Gaya F., Cabanas F., Burgueros M., Quero J. (1995). Multiple organ involvement in perinatal asphyxia. J. Pediatr..

[26] Hankins G.D., Koen S., Gei A.F., Lopez S.M., Van Hook J.W., Anderson G.D. (2002). Neonatal organ system injury in acute birth asphyxia sufficient to result in neonatal encephalopathy. Obs. Gynecol..

[27] Szakmar E., Jermendy A., El-Dib M. (2019). Respiratory management during therapeutic hypothermia for hypoxic-ischemic encephalopathy. J. Perinatol..

[28] Gaies M.G., Jeffries H.E., Niebler R.A., Pasquali S.K., Donohue J.E., Yu S., Gall C., Rice T.B., Thiagarajan R.R. (2014). Vasoactive-inotropic score is associated with outcome after infant cardiac surgery: An analysis from the Pediatric Cardiac Critical Care Consortium and Virtual PICU System Registries. Pediatr. Crit. Care Med..

[29] Miletic K.G., Spiering T.J., Delius R.E., Walters H.L., Mastropietro C.W. (2015). Use of a novel vasoactive-ventilation-renal score to predict outcomes after paediatric cardiac surgery. Interact. Cardiovasc. Thorac. Surg..

[30] Alam S., Akunuri S., Jain A., Mazahir R., Hegde R. (2018). Vasoactive-ventilation-renal score in predicting outcome postcardiac surgery in children. Int. J. Crit. Illn. Inj. Sci..

[31] Miletic K.G., Delius R.E., Walters H.L., Mastropietro C.W. (2016). Prospective Validation of a Novel Vasoactive-Ventilation-Renal Score as a Predictor of Outcomes After Pediatric Cardiac Surgery. Ann. Thorac. Surg..

[32] Scherer B., Moser E.A., Brown J.W., Rodefeld M.D., Turrentine M.W., Mastropietro C.W. (2016). Vasoactive-ventilation-renal score reliably predicts hospital length of stay after surgery for congenital heart disease. J. Thorac. Cardiovasc. Surg..

[33] Cashen K., Costello J.M., Grimaldi L.M., Narayana Gowda K.M., Moser E.A.S., Piggott K.D., Wilhelm M., Mastropietro C.W. (2018). Multicenter Validation of the Vasoactive-Ventilation-Renal Score as a Predictor of Prolonged Mechanical Ventilation After Neonatal Cardiac Surgery. Pediatr. Crit. Care Med..

[34] Chock V.Y., Frymoyer A., Yeh C.G., Van Meurs K.P. (2018). Renal Saturation and Acute Kidney Injury in Neonates with Hypoxic Ischemic Encephalopathy Undergoing Therapeutic Hypothermia. J. Pediatr..

[35] Askenazi D.J., Ambalavanan N., Goldstein S.L. (2009). Acute kidney injury in critically ill newborns: What do we know? What do we need to learn?. Pediatr. Nephrol..

[36] Selewski D.T., Jordan B.K., Askenazi D.J., Dechert R.E., Sarkar S. (2013). Acute kidney injury in asphyxiated newborns treated with therapeutic hypothermia. J. Pediatr..

[37] Sarkar S., Askenazi D.J., Jordan B.K., Bhagat I., Bapuraj J.R., Dechert R.E., Selewski D.T. (2014). Relationship between acute kidney injury and brain MRI findings in asphyxiated newborns after therapeutic hypothermia. Pediatr. Res..

[38] Kidney Disease: Improving Global Outcomes (KDIGO) CKD-MBD Work Group (2009). KDIGO clinical practice guideline for the diagnosis, evaluation, prevention, and treatment of Chronic Kidney DiseaseMineral and Bone Disorder (CKD-MBD). Kidney Int. Suppl..

[39] Sweetman D.U. (2017). Neonatal acute kidney injury-Severity and recovery prediction and the role of serum and urinary biomarkers. Early Hum. Dev..

[40] Choudhary M., Sharma D., Dabi D., Lamba M., Pandita A., Shastri S. (2015). Hepatic dysfunction in asphyxiated neonates: Prospective case-controlled study. Clin. Med. Insights Pediatr..

[41] Muniraman H., Gardner D., Skinner J., Paweletz A., Vayalakkad A., Chee Y.H., Clifford C., Sanka S., Venkatesh V., Curley A. (2017). Biomarkers of hepatic injury and function in neonatal hypoxic ischemic encephalopathy and with therapeutic hypothermia. Eur. J. Pediatr..

[42] Leung D.H., Khan M., Minard C.G., Guffey D., Ramm L.E., Clouston A.D., Miller G., Lewindon P.J., Shepherd R.W., Ramm G.A. (2015). Aspartate aminotransferase to platelet ratio and fibrosis-4 as biomarkers in biopsy-validated pediatric cystic fibrosis liver disease. Hepatology.

[43] Chen S., Yin T., Li L., Diao M., Huang T. (2023). Development and validation of non-invasive models in predicting advanced fibrosis of choledochal cyst. Pediatr. Surg. Int..

[44] Okamoto C., Tsukamoto O., Hasegawa T., Hitsumoto T., Matsuoka K., Amaki M., Kanzaki H., Izumi C., Takashima S., Ito S. (2022). Candidate Screening for Heart Failure With Preserved Ejection Fraction Clinic by Fib-4 Index From Subclinical Subjects. Gastro Hep Adv..

[45] Eto F., Nezu T., Aoki S., Kuzume D., Hosomi N., Maruyama H. (2024). Liver fibrosis index is associated with functional outcome among acute ischemic stroke patients. J. Stroke Cerebrovasc. Dis..

[46] Wang Z., Li G., Huang R., Chang L., Gong C., Chen K., Wang L. (2023). Prognostic value of fibrosis-5 index combined with C-reactive protein in patients with acute decompensated heart failure. BMC Cardiovasc. Disord..

[47] Gharipour A., Razavi R., Gharipour M., Mukasa D. (2020). Lactate/albumin ratio: An early prognostic marker in critically ill patients. Am. J. Emerg. Med..

[48] Guo W., Zhao L., Zhao H., Zeng F., Peng C., Guo W., Yan H. (2021). The value of lactate/albumin ratio for predicting the clinical outcomes of critically ill patients with heart failure. Ann. Transl. Med..

[49] Kabra R., Acharya S., Shukla S., Kumar S., Wanjari A., Mahajan S., Gaidhane S.A., Bhansali P.J., Wasnik P., Gaidhane S.A. (2023). Serum lactate-albumin ratio: Soothsayer for outcome in sepsis. Cureus.

[50] Nishioka N., Kobayashi D., Izawa J., Irisawa T., Yamada T., Yoshiya K., Park C., Nishimura T., Ishibe T., Yagi Y. (2021). Association between serum lactate level during cardiopulmonary resuscitation and survival in adult out-of-hospital cardiac arrest: A multicenter cohort study. Sci. Rep..

[51] Shi X., Zhong L., Lu J., Hu B., Shen Q., Gao P. (2024). Clinical significance of the lactate-to-albumin ratio on prognosis in critically ill patients with acute kidney injury. Ren. Fail..

[52] Wang R., He M., Qu F., Zhang J., Xu J. (2022). Lactate albumin ratio is associated with mortality in patients with moderate to severe traumatic brain injury. Front. Neurol..

[53] Sai I.N., Prasad R. (2022). Assessing the prognostic value of crp/albumin ratio and lactate/albumin ratio in critically ill patients. J. Assoc. Physicians India..

[54] Zhu X., Xue J., Liu Z., Dai W., Xu H., Zhou Q., Zhao S., Zhou Q., Chen W. (2021). The lactate/albumin ratio predicts mortality in critically ill patients with acute kidney injury: An observational multicenter study on the eICU database. Int. J. Gen. Med..

[55] Arı H.F., Keskin A., Arı M., Aci R. (2024). Importance of lactate/albumin ratio in pediatric nosocomial infection and mortality at different times. Future Microbiol..

[56] Aygün F., Durak C., Varol F., Çokuğraş H., Camcıoğlu Y. (2020). The Lactate/Albumin Ratio is an Effective Predictor for Mortality in Critically Ill Children. Türkiye Çocuk Hast. Derg..

[57] Sweetman D.U., Molloy E.J. (2013). Biomarkers of acute kidney injury in neonatal encephalopathy. Eur. J. Pediatr..

[58] Sweetman D.U., Onwuneme C., Watson W.R., O’Neill A., Murphy J.F., Molloy E.J. (2016). Renal function and novel urinary biomarkers in infants with neonatal encephalopathy. Acta Paediatr..

